# Measuring malevolent character: Data using the Swedish version of Jonason's Dark Triad Dirty Dozen

**DOI:** 10.1016/j.dib.2017.08.020

**Published:** 2017-08-31

**Authors:** Danilo Garcia, Patricia Rosenberg, Shane MacDonald, Christine Räisänen, Max Rapp Ricciardi

**Affiliations:** a Blekinge Center of Competence, Blekinge County Council, Karlskrona, Sweden; b Department of Psychology, University of Gothenburg, Gothenburg, Sweden; c Network for Empowerment and Well-Being, Sweden; d Department of Psychology, Lund University, Lund, Sweden; e Center for Health and Medical Psychology (CHAMP), Örebro, Sweden; f Psychological Links of Unique Strengths (PLUS), Stockholm, Sweden; g Civil and Environmental Engineering, Construction Management, Chalmers University of Technology, Gothenburg, Sweden

## Abstract

The data include responses to the Swedish version of a brief questionnaire used to operationalize the Dark Triad of malevolent character: Machiavellianism, narcissism, and psychopathy. The data was collected among 342 Swedish university students and white-collar workers (see Garcia et al. (2017) [1]). In this article, we include the Swedish version of Jonason's Dark Triad Dirty Dozen questionnaire. The data is available, SPSS and cvs file, as supplementary material in this article. Additionally, we also provide the scoring key as SPSS syntax file.

**Specifications Table**

**Table:** 

Subject area	*Psychology*
More specific subject area	*Dark Triad, Machiavellianism, Malevolent Character, Narcissism, Personality, Psychopathy, Sweden*
Type of data	*Swedish version of Jonason's Dark Triad Dirty Dozen, SPSS/cvs file, and scoring key as SPSS syntax file.*
How data was acquired	*Online survey (distributed through Qualtrics)*
Data format	*Analyzed*
Experimental factors	*The study had a cross-sectional design*
Experimental features	*The main variables were gender, Machiavellianism, narcissism, and psychopathy*
Data source location	*Sweden*
Data accessibility	*Data is within this article and as supplementary material*

**Value of the data**

• This data can be employed for individual statistical analysis and meta-analysis.
• The data was collected in Sweden and has cultural diversity value.
• The questionnaire can be used for data collection among Swedish speaking participants.

## 1 Data

The Swedish version of Jonason's Dark Triad Dirty Dozen questionnaire was answered by 342 individuals.

## 2 Experimental design, materials and methods

### 2.1 Participants

The data were collected among 342 university students and white-collar workers with an age range between 19–65 (166 males, 175 females, and 1 who did not report gender).

### 2.2 Questionnaire

Jonason's Dark Triad Dirty Dozen [2] is a 12-item self-report questionnaire designed to measure the three Dark Triad traits: Machiavellianism, narcissism, and psychopathy. Participants are asked to rate how much they agreed (1 = *Strongly disagree*; 7 = *Strongly agree*) with statements such as: ‘‘I tend to manipulate others to get my way’’ (Machiavellianism), ‘‘I tend to lack remorse’’ (psychopathy), and ‘‘I tend to want others to admire me’’ (narcissism). See Appendix A for the questionnaire in Swedish and English. Items were averaged to create each dark trait. The data presented here obtained acceptable *Cronbach's alphas* for all three dark traits in the whole sample: .74 for Machiavellianism, .78 for narcissism, .63 for psychopathy, and .80 for the dark triad core (i.e., the sum of participant's scores in the three dark traits). See Table 1 for means and standard deviations for males, females and the whole sample.

**Table 1:** Means, standard deviations (SD), and Cronbach's Alpha for males, females, and the whole sample.

	Males (n = 166)			Females (n = 175)			Total (N = 341)		
	Mean	SD	Cronbach's Alpha	Mean	SD	Cronbach's Alpha	Mean	SD	Cronbach's Alpha
Machiavellianism	2.67	1.09	.73	2.76	1.15	.75	2.72	1.12	.74
Psychopathy	2.22	1.12	.60	1.81	1.00	.65	2.01	1.08	.63
Narcissism	2.98	1.19	.76	3.22	1.26	.81	3.11	1.23	.78
Dark Triad Core	2.62	0.80	.80	2.60	0.80	.80	2.61	0.80	.80

Note: One individual did not report gender, thus, her/his scores are not reported here. The dark triad core is the sum of participant's scores in the three dark traits.

### 2.3 Statistical issues and caveats

The validity of Jonason's Dark Triad Dirty Dozen has been criticized elsewhere [3]. Additionally, for the Swedish version of the Dark Triad Dirty Dozen we opted to use a 7-point Likert scale as recommended by Jonason in some of his studies [4]. However, in other studies, Jonason has used a 5-point Likert scale [5], [6] or even a 9-point Likert scale [7]. Hence, it is difficult to compare samples. Finally, the results of our confirmatory factor analysis [1] showed that, despite having good correspondence to the English version, there was mixed evidence for model fit.

## Figures and Tables

**Table:** 

Trait	Item No.	English	Swedish	Håller inte alls med	Håller inte med	Håller delvis inte med	Varken eller	Håller med delvis	Håller med	Håller med helt
MACHIAVELLIANISM	1	I tend to manipulate others to get my way.	Jag tenderar att manipulera andra för att få det jag vill.	1	2	3	4	5	6	7
	2	I have used deceit or lied to get my way.	Jag har bedragit eller ljugit för att få det jag vill.	1	2	3	4	5	6	7
	3	I have use flattery to get my way.	Jag har använt mig av smicker för att få min vilja igenom.	1	2	3	4	5	6	7
	4	I tend to exploit others towards my own end.	Jag tenderar att utnyttja andra för att nå mina egna mål.	1	2	3	4	5	6	7
PSYCHOPATHY	5	I tend to lack remorse.	Jag tenderar att sakna ånger.	1	2	3	4	5	6	7
	6	I tend to be unconcerned with the morality of my actions.	Jag tenderar att inte bry mig om moralen i mina handlingar.	1	2	3	4	5	6	7
	7	I tend to be callous or insensitive.	Jag tenderar att vara kall eller okänslig.	1	2	3	4	5	6	7
	8	I tend to be cynical.	Jag tenderar att vara cynisk.	1	2	3	4	5	6	7
NARCISSISM	9	I tend to want others to admire me.	Jag tenderar att vilja att andra beundrar mig.	1	2	3	4	5	6	7
	10	I tend to want others to pay attention to me.	Jag tenderar att vilja att andra uppmärksammar mig.	1	2	3	4	5	6	7
	11	I tend to seek prestige or status.	Jag tenderar att söka prestige och status.	1	2	3	4	5	6	7
	12	I tend to expect special favors from others.	Jag tenderar att förvänta mig speciella tjänster av andra.	1	2	3	4	5	6	7

## Appendix 1

### Dark Triad Dirty Dozen^1^

#### Instruktion

Var vänlig och indikera i vilken utsträckning du håller med respektive påstående. Försök att vara så öppen och ärlig i dina svar som möjligt.
